# Field testing the alternative criteria for tension-type headache proposed in the third beta edition of the international classification of headache disorders: results from the Korean headache-sleep study

**DOI:** 10.1186/1129-2377-15-28

**Published:** 2014-05-13

**Authors:** Min Kyung Chu, Soo-Jin Cho, Jae-Moon Kim, Sung-Hee Hwang

**Affiliations:** 1 Department of Neurology, Sacred Heart Hospital, Hallym University College of Medicine, Anyang, Korea; 2 Department of Neurology, Dongtan Sacred Heart Hospital, Hallym University College of Medicine, Hwaseong, Korea; 3 Department of Neurology, Chungnam National University, College of Medicine, Daejeon, Korea; 4 Department of Neurology, Kangnam Sacred Heart Hospital, Hallym University College of Medicine, Seoul, Korea

**Keywords:** Classification, Criteria, Headache, Migraine, Probable migraine, Tension-type headache

## Abstract

**Background:**

According to the third beta edition of the International Classification of Headache Disorder (ICHD-3 beta), the diagnostic criteria for tension-type headache (TTH) might lead to the inclusion of individuals with headaches showing migrainous features. To better define TTH, the alternative diagnostic criteria were proposed in the appendix of ICHD-3 beta. This study attempted to test the alternative criteria for diagnosis of TTH proposed in ICHD-3 beta in a population-based sample from Korea.

**Methods:**

We selected participants from the Korean population aged 19–69 years using stratified random sampling and evaluated them by interview using a questionnaire designed to identify headache type, headache characteristics, and psychiatric comorbidities.

**Results:**

Of the 2,762 participants, 586 (21.3%) were diagnosed as having TTH using the standard criteria. Among these, 238 (40.6%) were also classified as having TTH using the alternative criteria. All 238 TTH subjects first diagnosed as having TTH by the alternative criteria were also classified as having TTH by the standard criteria. If the standard criteria were not applied, the remaining 348 patients were subclassified as having probable migraine (115, 19.6%) and unclassified headache (233, 39.7%). Compared with subjects diagnosed with TTH using the standard criteria, those diagnosed using the alternative criteria were less likely to demonstrate unilateral, pulsating headache, which is aggravated by movement, photophobia, phonophobia, and osmophobia.

**Conclusion:**

Using the alternative criteria, less than half of the subjects with TTH according to the standard criteria were classified as having TTH. All the subjects with TTH by the alternative criteria were classified as having TTH by the standard criteria. This study also demonstrated that subjects diagnosed with TTH using the standard criteria could include people with headaches showing migrainous features.

## Background

Tension-type headache (TTH) is the most prevalent headache disorder in the general population, mostly ranging from 20% to 60% [1-3]. Although symptoms of TTH are usually mild, some individuals with TTH experience disability or impaired quality of life [4,5]. Owing to its high prevalence, TTH causes large amounts of socioeconomic burden due to absenteeism or reduced effectiveness in the workplace, school, or home [3,6]. However, little attention has been paid to TTH by health authorities, practitioners, and pharmaceutical companies.

Currently, there are no helpful diagnostic tools or key features for the diagnosis of TTH. The diagnosis of TTH completely relies on clinical symptoms, which are less specific than those of migraine or other headaches. The diagnosis of TTH is mainly based on the absence of typical features found in other types of headache. Therefore, TTH has been considered to be a featureless headache [7].

The first edition of the International Classification of Headache Disorder (ICHD-1) described the general criteria for TTH based on five features: total attack number, headache duration, pain characteristics, associated symptoms, and non-other headache. TTH was divided into episodic tension-type headache (ETTH) and chronic tension-type headache (CTTH) depending on the number of days with TTH [8]. The general criteria for TTH in the second edition of International Classification of Headache Disorder (ICHD-2) were nearly identical to those of the first edition [9]. In June 2013, the third beta edition of ICHD (ICHD-3 beta) was proposed, and the general criteria for TTH were essentially the same as those of the first and the second edition [10].

The diagnostic difficulty often encountered among the primary headache disorders is to discriminate between TTH and migraine. Tightened diagnostic criteria for TTH, the alternative criteria, were proposed in the hope of excluding migraine that phenotypically resembled TTH in the Appendix of ICHD-2 and ICHD-3 beta [9,10]. They define more strictly a core syndrome of TTH. Such stricter criteria may increase specificity and reduce the sensitivity of the criteria. The Classification Committee of International Headache Society recommended comparisons between patients diagnosed according to each set of criteria [10]. However, the alternative criteria have not yet been tested.

The present study was undertaken to test the alternative criteria by comparing the results produced by the standard criteria with the results produced by the alternative criteria, using data from the Korean Headache-Sleep Study, a population-based sample in Korea. In addition, we compared sociodemographics, clinical characteristics, medication-overuse headache (MOH) and psychiatric comorbidity of all subjects diagnosed with TTH.

## Methods

This study provides a nation-wide, cross-sectional survey of headache in the Korean population. Trained interviewers conducted structured interviews using a questionnaire to diagnose headache disorders in adults aged 19–69 years. The interview included questions on the symptoms and impact of headache. Socioeconomic, demographic, and geographic factors for the participants were also evaluated. This study was carried out from November 2011 to January 2012. The Institutional Review Board and the ethics committee of Hallym University Sacred Heart Hospital approved the study.

### Target area

The estimated total population of Korea in 2010 was 48,580,293, of which approximately 32,356,747 people were aged 19–69 years, based on the data from the 2010 Population and Housing Census conducted by the National Statistical Office [11]. Korea is geographically sectored into 15 administrative divisions (“do”) except Jeju-do. Each of these is further divided into “si,” “gun,” or “gu,” which form the basic administrative units. This study included all the Korean territories except Jeju-do. For this study, we classified seven metropolitan “si” areas (Seoul, Busan, Daegu, Incheon, Gwangju, Daejeon, and Ulsan) as “large city,” other “si” areas as “medium-to-small city,” and “gun” areas as “countryside.”

### Sampling

To obtain epidemiological data for common primary headache disorders, we aimed to sample 2,750 individuals based on the population structure. We adopted a two-stage systematic random sampling method. The 15 administrative divisions were designated as the primary sampling units. We assigned appropriate sample numbers to primary sampling units according to their population distributions. In the second stage, we further selected representative basic administrative units (si, gun, and gu) from each primary sampling unit. Overall, 60 representative basic administrative units were selected for this study. For each representative basic administrative unit, we assigned a target sample size based on age, gender, and occupation. The estimated sampling error for our study is ±1.8% with 95% confidence interval (CI) (Table 1) [12].

**Table 1:** Sociodemographic-distribution of all survey participants, the total Korean population and of cases identified as tension-type headache

	**Sample number, N (%)**	**Total population, N (%)**	***p*-value**	**Individuals with tension-type headache according to the standard criteria, Number, adjusted prevalence (95% CI)**	**Individuals with tension-type headache according to the alternative criteria, Number, adjusted prevalence (95% CI)**	***p*-value**	**AOR (95% CI)**
Gender							
Men	1377 (49.2^a^)	17,584,365 (50.6)	0.84	272, 19.8 (17.7-22.0^b^)	125, 9.0 (7.5-10.6^b^)	REFERENCE	REFERENCE
Women	1385 (50.8^a^)	17,198,350 (49.4)		314, 22.7 (20.5-24.9^b^)	113, 8.2 (6.8-9.7^b^)	0.117	0.781 (0.573-1.064^c^)
Age							
19-29	542 (20.0^a^)	7,717,947 (22.2)	0.99	119, 22.0 (18.5-25.5^b^)	45, 8.4 (6.0-10.7^b^)	REFERENCE	REFERENCE
30-39	604 (21.3^a^)	8,349,487 (24.0)		127, 21.2 (17.9-24.5^b^)	47, 7.8 (5.6-9.9^b^)	0.962	0.988 (0.610-1.601^c^)
40-49	611 (22.5^a^)	8,613,110 (24.8)		131, 21.4 (18.2-24.7^b^)	57, 9.4 (7.1-11.6^b^)	0.422	1.211 (0.759-1.932^c^)
50-59	529 (18.4^a^)	6,167,505 (17.7)		107, 20.1 (16.7-23.7^b^)	50, 9.4 (6.9-12.0^b^)	0.315	1.325 (0.765-2.293^c^)
60-69	476 (17.8^a^)	3,934,666 (11.3)		102, 21.7 (18.1-25.3^b^)	39, 8.2 (5.7-10.6^b^)	0.978	0.991 (0.529-1.857^c^)
Size of residential area							
Large city	542 (20.0^a^)	16,776,771 (48.2)	0.90^b^	256, 20.1 (17.9-22.3^b^)	105, 8.2 (6.7-9.7^b^)	REFERENCE	REFERENCE
Medium-to-small city	604 (21.3^a^)	15,164,345 (43.6)		251, 20.8 (18.5-23.1^b^)	99, 8.2 (6.7-9.8^b^)	0.817	0.962 (0.694-1.334^d^)
Rural area	611 (22.5^a^)	2,841,599 (8.2)		79, 29.2 (23.8-34.6^b^)	34, 12.3 (8.4-16.2^b^)	0.837	1.050 (0.658-1.675^d^)
Educational level							
Middle school or less	446 (16.5^a^)	6,147,782 (19.0)	0.94	109, 24.7 (20.7-27.7^b^)	49, 11.1 (8.2-13.9^b^)	REFERENCE	REFERENCE
High school	1218 (43.8^a^)	14,172,255 (43.8)		249, 20.5 (18.2-22.8^b^)	92, 7.5 (6.0-9.0^b^)	0.286	0.759 (0.458-1.259^d^)
College or more	1005 (38.7^a^)	1,2036,710 (37.2)		224, 21.0 (18.5-23.4^b^)	97, 9.1 (7.3-10.8^b^)	0.817	0.935 (0.528-1.656^d^)
Total	2,762 (100.0^a^)	32,356,747 (100.0)		586, 21.3 (19.8-22.8^b^)	238, 8.6 (7.6-9.7^b^)		

AOR: adjusted odds ratio.

^a^Age- and gender-adjusted prevalence.

^b^Compared gender, age group, size of residential area, and educational level distributions between the sample of the present study and total population.

^c^Adjusted for size of residential area and educational level.

^d^Adjusted for size of age and gender.

### Questionnaire

The questionnaire comprised three parts. The first part assessed demographic and socioeconomic characteristics (9 questions). The second part established a headache profile, which was designed to comply with ICHD-2 (13 questions). The third part was composed of questions about mood disturbances. We included the Headache Impact Test-6 questionnaire (HIT-6) to evaluate the impact of headache on the quality of life.

### Survey

The study objectives and methods were explained to our interviewers, who then interviewed a specific number of participants (aged 19–69 years) on the basis of age, gender and profession distribution. Before beginning face-to-face interviews, the interviewers were provided with the following information: (1) the aims of the study, (2) the meaning of each question, (3) the importance of checking participants’ responses, and (4) other details regarding how to properly conduct an interview. All the interviewers were employed by Gallup Korea and had previous social survey interviewing experience. The survey was conducted by door-to-door visits and face-to-face interviews. To avoid interest bias for headache, interviewers recruited participants in interview for “health status” rather than headache.

### Case definition of TTH according to the standard criteria

The diagnosis of TTH according to standard criteria was based on the ICHD-3 beta criteria for ETTH (code 2.1 and 2.2) and CTTH (code 2.3). This required the experience of 10 or more episodes in a lifetime, with each attack lasting from 30 min to 7 days and accompanied by two or more of the following four pain characteristics: bilateral location, non-pulsating quality, mild-to-moderate intensity, and without aggravation by routine physical activity. In addition, the attacks must have been associated with both of the following: no nausea or vomiting, and no more than one of either photophobia or phonophobia [10]. The diagnosis of TTH using the questionnaire was validated with 86.2% sensitivity and 75.5% specificity by comparing it to the doctors’ diagnosis obtained from in an additional telephone interview for the previous study [13].

### Case definition of TTH according to the alternative criteria

The diagnosis of TTH according to the alternative criteria was established using the criteria listed in the appendix of ICHD-3 beta (code A2). We included only subjects who had experienced more than 10 episodes in a lifetime, with each attack lasting from 30 min to 7 days accompanied by at least three of the following four pain characteristics: bilateral location, non-pulsating quality, mild-to-moderate intensity, and without aggravation by routine physical activity. In addition, the attacks should not have been associated with nausea, vomiting, photophobia, or phonophobia [10]. Assessment of TTH according to the alternative criteria was conducted in all the participants of this study.

### Case definition of infrequent ETTH, frequent ETTH and CTTH

Tension-type headache by the standard criteria and the alternative criteria was subclassified as infrequent ETTH, frequent ETTH and CTTH based on the headache days per month on average: <1 days per month for infrequent ETTH; 1–14 days per month for frequent ETTH; and ≥15 days per month for CTTH.

### Case definition of MOH

A diagnosis of MOH was based on the criteria for MOH criteria in ICHD-3 beta (code 8.2) [10]. A participant with headache occurring ≥15 days per month was diagnosed with MOH if he/she reported regularly overusing acute/symptomatic treatment drugs that were defined in either criteria 1 or 2 for more than 3 months (criterion 1: ergotamine, triptans, opioids, or a combination of analgesics, triptans, or analgesic opioids ≥10 days/month for >3 months; criterion 2: simple analgesics or any combination of ergotamine, triptans, or analgesics opioids ≥15 days/month without the overuse of any single class alone).

### Case definition of probable migraine

A diagnosis of probable migraine (PM) was based on the A–D criteria for migraine without aura (code 1.1) in the ICHD-3 beta (A: five or more attacks in a lifetime; B: attack duration of 4 to 72 h; C: any two of the following four typical headache characteristics: unilateral pain, pulsating quality, moderate-to-severe intensity, and aggravation by routine physical activity); D: attacks associated with at least one of the following conditions–nausea or vomiting or both photophobia and phonophobia. These criteria were the same as those specified in ICHD-2. If a participant’s response met all criteria except for one, s/he was identified as having PM [10].

### Case definition of anxiety

We used the Goldberg Anxiety Scale (GAS) for the diagnosis of anxiety. GAS is composed of four screening questions and five complementary questions [14]. If a participant answered positively ≥2 of the first 4 screening questions, and ≥5 of all GAS questions, s/he was assigned a diagnosis of anxiety. The GAS was validated for the diagnosis of anxiety using the Korean language with 82.0% sensitivity and 94.4% specificity [15].

### Case definition of depression

The Patient Health Questionnaire-9 (PHQ-9) was used for the diagnosis of depression [16]. If a participant’s PHQ-9 score was ≥10, s/he was assigned as having depression. PHQ-9 was validated in the Korean language with 81.1% sensitivity and 89.9% specificity [17].

### Analyses

The 1-year prevalence with 95% confidence interval is presented as the number of cases per 100 persons diagnosed according to the standard criteria or the alternative criteria. Age- and gender-specific prevalence rates were also calculated. The results were analyzed using statistical software SPSS 21.0 (IBM, Armonk, NY, USA). Student’s *t*-test and Chi-square test were used for comparisons when appropriate. The level of statistical significance was set at *p* < 0.05.

We examined odds ratios (ORs; 95% CIs) for TTH diagnosed according to the alternative or standard criteria, using multivariate logistic regression analyses, with sociodemographic variables (gender, age, size of residential area and educational level) as covariates.

As with most survey sampling designs, missing data resulting from non-response occurred in several variables. The data reported are based on the available data. Sample sizes of some variables diverge from the sample size of n = 2,762 because of non-responses on that particular variable. Imputation techniques were not employed to minimize non-response effects [18].

## Results

### Samples

**Figure 1:**
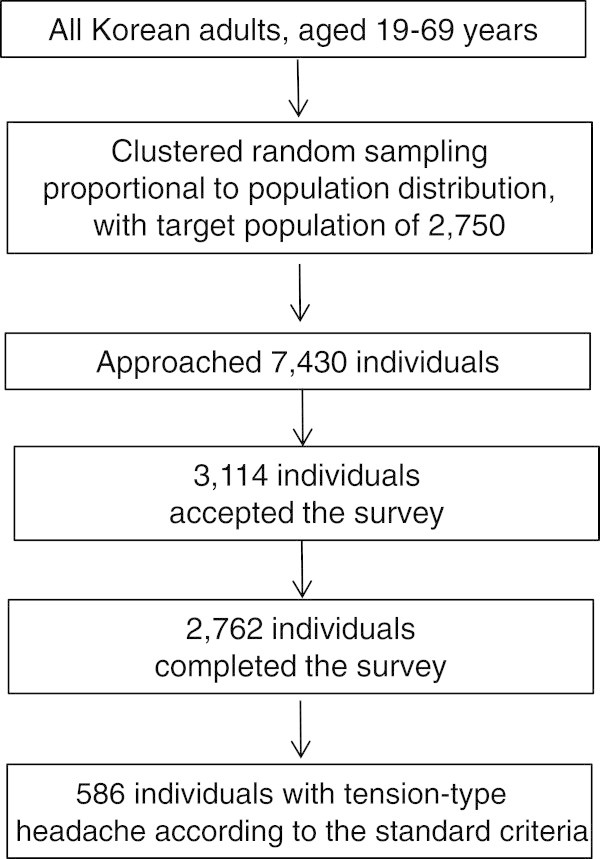
Flow chart depicting the participation of subjects in the Korean Headache-Sleep Study.

Of the 7,430 individuals approached by our 76 interviewers, 3,114 agreed to participate in the survey. Of these 3,114 participants, 352 individuals suspended the interview, leaving 2,762 subjects who completed the survey (cooperation rate of 37.2%; Figure 1). The distributions of age, gender, size of residential area, and educational level across our samples were not significantly different from those in the total Korean population (Table 1).

### The prevalence of TTH according to the standard criteria and the alternative criteria

Of the 2,762 participants, 1,299 (47.2%) subjects had at least one attack of headache, and 586 subjects (21.3%, 95% CI 19.8–22.8%) were classified as having TTH according to the standard criteria during the previous year. In contrast, 238 (8.6%, 95% CI 7.6–9.7%) subjects were classified as having TTH according to the alternative criteria.

### Classification of subjects with TTH according to the standard criteria and the alternative criteria

**Figure 2:**
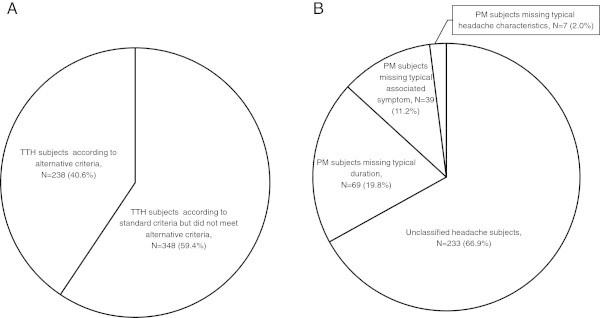
**Distribution of tension-type headache subjects according to standard criteria (A) and tension-type headache subjects according to standard criteria missing alternative criteria (B).** PM: probable migraine; TTH: tension-type headache.

Of the 586 subjects diagnosed with TTH using the standard criteria, 238 (40.6%) subjects were also classified as having TTH according to the alternative criteria (Figure 2-A). The remaining 348 subjects were subclassified as having PM (*n* = 115, 33.1%) and unclassified headache (*n* = 233, 66.9%) (Figure 2-B). Among the 115 subjects with PM, 69 subjects did not meet the criterion for typical time duration, 39 subjects for typical associated symptoms, and 7 subjects for typical headache characteristics.

### Sociodemographic characteristics of subjects with TTH according to the standard criteria and the alternative criteria

The distributions of age, gender, size of residential area, and educational level for subjects with TTH were not significantly different between those diagnosed using the standard criteria and the alternative criteria (Table 1).

### Clinical characteristics and impact of TTH according to the standard criteria and the alternative criteria

Subjects with TTH defined by the alternative criteria had headaches showing a more bilateral location, non-pulsating quality, and non-aggravation by movement, and less osmophobia compared with those defined by the standard criteria. However, the latter showed photophobia and phonophobia, which were not seen in the former. Although the proportions of chronic TTH between two criteria were not significantly different, infrequent episodic TTH was more prevalent when using the alternative criteria and frequent episodic TTH was more prevalent when using the standard criteria. The HIT-6 score of subjects with TTH according to the alternative criteria was lower compared with that of subjects with TTH according to the standard criteria. Headache severity, MOH, anxiety, and depression of subjects with TTH were not significantly different for the two sets of criteria (Table 2).

**Table 2:** Clinical characteristics of subjects with tension-type headache according to the standard criteria and the alternative criteria

		**Subjects with tension-type headache according to the standard criteria, N = 586, N (%)**	**Subjects with tension-type headache according to the alternative criteria, N = 238, N (%)**	**P-value**^**a**^
Headache characteristics	Bilateral location	383 (65.4)	189 (79.8)	<0.001
	Non-pulsating quality	235 (40.1)	127 (53.1)	0.001
	Mild-to-moderate pain intensity	576 (98.1)	235 (98.7)	0.540
	Non-aggravation by movement	463 (78.9)	234 (98.3)	<0.001
Associated symptoms	Nausea	0 (0.0)	0 (0.0)	
	Vomiting	0 (0.0)	0 (0.0)	
	Photophobia	48 (8.1)	0 (0.0)	<0.001
	Phonophobia	190 (32.6)	0 (0.0)	<0.001
	Osmophobia	99 (17.0)	15 (6.3)	<0.001
Infrequent episodic TTH	<1 headache day per month	335 (57.2)	158 (66.4)	0.014
Frequent episodic TTH	1-14 headache days per month	235 (40.1)	71 (29.8)	0.006
Chronic TTH	≥15 headache days per month	16 (2.7)	9 (3.8)	0.418
Headache severity	Visual analogue score (VAS)	4.4 ± 1.8	4.1 ± 1.9	0.079
Impact of headache	HIT-6 score	43.9 ± 6.5	42.6 ± 6.2	0.006
Medication-overuse headache		3 (0.5)	1 (0.4)	0.864
Psychiatric comorbidity	Anxiety	55 (9.3)	13 (5.4)	0.064
	Depression	110 (18.7)	36 (15.1)	0.216

HIT-6: headache impact test-6; TTH: tension-type headache.

^a^Compared between individuals with tension-type headache according to standard criteria and individuals with tension-type headache according to alternative criteria.

## Discussion

The key findings in the present study are as follows: 1) The 1-year prevalence of TTH meeting the standard criteria and the alternative criteria were 21.3% and 8.6% in the Korean population sample, respectively; 2) Among subjects meeting the standard criteria for TTH, 40.6% were also classified as having TTH according to the alternative criteria, and all subjects with TTH by the alternative criteria were classified as having TTH by the standard criteria; 3) Tension-type headache subjects according to the standard criteria could include people with headaches showing migrainous features.

The prevalence of TTH in Asian countries, ranged from 21.7% to 30.8%, and was similar or somewhat lower compared with other regions [13,19-23]. The 1-year prevalence of TTH according to the standard criteria in the present study was similar to, or somewhat lower than, those in previous Asian studies. The wide range of prevalence rates in these studies might be related to methodological differences between the studies, discrepancies in the application of criteria in different languages, differences in sociocultural backgrounds, and genetic factors.

Current diagnosis for TTH is based on the patient’s report on the characteristics of headache, owing to lack of biomarkers or definitive headache features [7]. The diagnostic criteria for TTH in ICHD-3 beta might result in inclusion of individuals with headaches showing unilateral pain, pulsatile qualities, moderate-to-severe intensity, and aggravation by movement. These four headache characteristics are considered migrainous and are included in the criteria for typical headache characteristics in ICHD-3 beta. The alternative criteria define typical characteristics more exactingly, and include fewer migrainous headache characteristics.

In addition to migrainous headache, photophobia, phonophobia, and osmophobia were reported in some subjects with TTH in the present study. These are considered symptoms of sensory hypersensitivity due to migraine [24-26]. Recent studies have revealed that this hypersensitivity is mediated by modulation of the trigeminovascular pathway by the brainstem, hypothalamus and cortex [27,28]. The presentation of sensory hypersensitivity symptoms in subjects with TTH suggests that some migrainous headaches may be classified as TTH or some subjects with TTH may have migrainous features. The presence of fewer migrainous features among subjects diagnosed with TTH using the alternative criteria may imply higher specificity of these criteria, compared to the standard criteria. In the present study, fewer than half of the subjects with TTH as per the standard criteria were classified as having TTH by the alternative criteria, and all subjects with TTH by the alternative criteria were classified as having TTH by the standard criteria. Strict diagnostic criteria, which permits unequivocal diagnosis, was preferred for clinical and research trials [29]. The alternative criteria may provide better definition of TTH for such purposes.

If the alternative criteria was applied instead of the standard criteria, approximately 1/3 of subjects with TTH were classified as having PM and the remaining 2/3 were as having unclassified headache in the present study. A high prevalence of unclassified headache suggests usefulness of the standard criteria especially assigning headache diagnosis for clinical practice and epidemiological studies and limitation of the alternative criteria for these purposes.

The association between psychiatric comorbidity and TTH is much weaker than that for migraine [30]. In the present study, the presence of anxiety and depression in subjects with TTH did not differ significantly between those that were diagnosed using either the standard criteria or the alternative criteria.

Although the response rate is not high, we used clustered random sampling pool of Gallup Korea, which was validated with sampling error of ±1.8% [12]. We included participants with non-headache sufferers in this survey and socio-demographic distributions of participants of our survey were similar to those of whole population of Korea (Table 1). The prevalence rates of tension-type headache in our survey was similar to those studies previously done in Korea and other Asian countries [2,13,19-22]. Use of reliable sampling method with low sampling error, similarity in socio-demographic distributions to total population of Korea and similarity in TTH prevalence rate to previous studies done in Korea and other Asian countries supported that our study reflected TTH status of Korean population properly.

This study has several limitations. Firstly, we tested the alternative criteria of TTH by comparing subjects between the alternative criteria and the standard criteria. Some subjects identified as TTH might not have the diagnosis of TTH in doctors’ interview. For better definition of headache, combination of a diagnostic diary and clinical interview was recommended [31]. However, as in most epidemiological studies handling a large sample size, keeping headache diary is very difficult and we could not use headache diary in the present study. Owing to lack of golden standard, we could not estimate the the sensitivity and the specificity of the alternative criteria in the present study. Further studies assessing headaches using both diagnostic diary and doctors’ interview would provide better definition of TTH. Secondly, we assigned only one possible headache diagnosis to each patient. A patient may have more than one type of headache, and TTH and migraine can often occur in the one patient [32,33]. If migraine exists in a subject with TTH, the clinical manifestations of TTH may be affected by the co-occurrence of migraine [34]. Further studies that acknowledge the co-existence of other headache types may provide better insight into the clinical characteristics and pathophysiology of TTH. Thirdly, although the present study was a population-based study with low sampling error, its statistical power was limited for examining subgroups. Thus, some conditions that did not reach statistical significance could merely be a reflection of the limited sample numbers.

The strength of this study lies in its large sample size, population-based format, low estimated sampling error, and use of a validated questionnaire for anxiety, depression, and TTH diagnosis using the standard criteria.

## Conclusions

Less than half of the subjects with TTH according to the standard criteria were classified as having TTH by the alternative criteria, and all the subjects with TTH by the alternative criteria were classified as having TTH by the standard criteria. This study also demonstrated that some patients with TTH might also have migrainous headaches. Further studies are needed to examine the physiological differences between headaches categorized as TTH using the standard criteria and the alternative criteria.

## Abbreviations

CTTH: Chronic tension-type headache; ETTH: Episodic tension-type headache; ICHD-1: The first edition of International Classification of Headache Disorders; ICHD-2: The second edition of International Classification of Headache Disorder; ICHD-3 beta: The third beta edition of the International Classification of Headache Disorders; GAS: Goldberg anxiety scale; MOH: Medication-overuse headache; PHQ-9: Patient health questionnaire-9; PM: Probable migraine; TTH: Tension-type headache.

## Competing interests

The authors declare that they have no conflicting interests.

## Authors’ contributions

SHH conceptualized and designed the study, analyzed the data, and wrote the manuscript. SJC conceptualized and designed the study, and wrote the manuscript. JMK conceptualized and collected the data. MKC conceptualized and designed the study, collected and analyzed the data, and wrote the manuscript. All authors read and approved the final manuscript.
